# Anaphylactic reactions induced by NSAID according to data of Vilnius University Hospital Santariskiu Klinikos

**DOI:** 10.1186/2045-7022-4-S3-P24

**Published:** 2014-07-18

**Authors:** Olesia Gaidej, Gabija Didziokaite, Neringa Buterleviciute, Viktorija Paltarackiene, Gintare Nakrosyte, Ana Kozlovska, Laura Malinauskiene, Violeta Kvedariene

**Affiliations:** 1 Vilnius Universiry, Centre of Pulmonology and Allergology, Lithuania; 2 Vilnius University, Faculty of Medicine, Lithuania

## 

Non-steroid anti-inflammatory drugs are one of easily accessible and most frequently used group of medications. Severe and life-threatening reactions are a challenge for doctors and nurses of all specialities.

The aim of this study is to investigate the frequency of severe and life-threatening reactions induced by NSAIDs.

## Methods

Patients with a history of drug allergies which were addressed to consultations by general practitioners were consulted in the Pulmonology and Allergology department in the period 2010-2013. The questionnaires and DPTs with the culprit drug or alternative medications were performed according to ENDA rules. Patients were divided into two groups depending on their clinical history and reaction severity.

## Results

244 patients addressed by general practitioners were consulted; female sex was predominant 68% (166 female). 444 different medications were mentioned in questionnaires. 182 (41%) of drugs were NSAIDs. Most frequent adverse reactions were observed doing to ASA for 16 (21%), for ketorolac in 11 (15%), for acetaminophen in 12 (15%) and for metamizol in 10 (12%) cases. Provocation tests for NSAIDs were performed on 60 patients. Drug provocation tests with the culprit drug were performed on 19 (32%) in the first group and in the second group for 41 (68%) patients, who had anaphylactic or life-threatening reactions, alternative medication has been chosen. 13 (22%) of tests were positive: 3 (5%) in the culprit drug provocation group, 10 (17%) in the alternative medication group. Immediate reactions were observed in 6 (10%) of all tested patients: anaphylaxis in 3 (5%), bronchospasm in 3 (5%). Two cases of anaphylaxis were observed in the first group, and only one in the second.

## Conclusions

Most frequent adverse reactions were observed using ASA, while ketorolac and acetaminophen took the second place. True hypersensitivity was diagnosed for one fifth of the patients. Anaphylactic reactions to NSAID hypersensitivity appeared on 10% of them.

